# In Vivo Regulation of Signal Transduction Pathways by Vitamin D Stabilizes Homeostasis of Human Immune Cells and Counteracts Molecular Stress

**DOI:** 10.3390/ijms241914632

**Published:** 2023-09-27

**Authors:** Julia Jaroslawska, Carsten Carlberg

**Affiliations:** 1 Institute of Animal Reproduction and Food Research, Polish Academy of Sciences, 10-748 Olsztyn, Poland; j.jaroslawska@pan.olsztyn.pl; 2 Institute of Biomedicine, School of Medicine, University of Eastern Finland, 70211 Kuopio, Finland

**Keywords:** vitamin D, vitamin D target genes, signal transduction, immune cells, HIF1, TNF, MAPK, NFκB

## Abstract

Vitamin D_3_ is a pre-hormone that regulates hundreds of target genes and dozens of physiological functions, including calcium homeostasis and the activity of the immune system, via its metabolite 1,25-dihydroxyvitamin D_3_, which is a high-affinity ligand for the transcription factor vitamin D receptor. In this study, we took advantage of data from the VitDHiD vitamin D_3_ intervention trial (25 healthy individuals) indicating that 442 protein-coding genes were significantly (false discovery rate < 0.05) up- or downregulated in peripheral blood mononuclear cells one day after taking a vitamin D_3_ bolus. Since more than half of the encoded proteins had “signaling” assigned as a primary biological function, we evaluated their involvement in signal transduction cascades included in the KEGG (Kyoto Encyclopedia of Genes and Genomes) database and found 88 of the vitamin D targets contributing to 16 different pathways. Eight of the pathways show an approximately even contribution of up- and downregulated genes, suggesting that the actions of vitamin D stabilize homeostasis of the physiological processes driven by the respective signaling cascades. Interestingly, vitamin D target genes involved in the signaling pathways of hypoxia-inducible factor 1 (HIF1), tumor necrosis factor (TNF), mitogen-activated protein kinases (MAPKs) and nuclear factor κB (NFκB) are primarily downregulated. This supports the observation that the physiological role of vitamin D in healthy individuals is to tone down certain processes rather than activate them. In conclusion, under in vivo conditions, vitamin D either alleviates the homeostasis of immune cells in healthy individuals or counteracts molecular responses to oxygen deprivation (HIF1), microbe infection (TNF), growth stimulation (MAPKs) and inflammation (NFκB).

## 1. Introduction

Signal transduction pathways are essential for the survival of tissues and cell types since they inform us about the status of the extracellular environment and induce appropriate molecular responses [1]. A typical signal transduction cascade is composed of an extracellular ligand, such as a cytokine, peptide hormone or growth factor; a membrane receptor specific for the respective ligand; intracellular adaptor proteins that transfer the activation status of the membrane receptor to cytosolic kinases and finally transcription factors or chromatin-modifying enzymes that are the targets of the activated kinases [2]. In this way, an extracellular signal is translated into changes in the transcriptome and epigenome of the stimulated cell. The sensitivity of the cell to the signal depends on the expression of the components of the respective signal transduction cascade [3]. For example, when a cell does not express the membrane receptor for a given cytokine, it cannot react to this signal [4]. Therefore, the up- and downregulation of genes encoding proteins involved in signaling processes has important consequences for the responsiveness of tissues and cell types.

In biomedical sciences, insight into the physiological role of a molecule or process is often obtained by studying its malfunction or absence. Accordingly, the molecule vitamin D_3_ (also called cholecalciferol) was named a vitamin more than 100 years ago because its deficiency can cause bone malformation disorders like rickets in children [5,6]. This indicates that vitamin D_3_ is essential for calcium homeostasis and, therefore, important for bone formation [7,8]. Vitamin D_3_ is the precursor to the nuclear hormone 1,25-dihydroxyvitamin D_3_ (1,25(OH)_2_D_3_) that acts as the high-affinity natural ligand of the transcription factor VDR (vitamin D receptor) [9]. The *VDR* gene is expressed in the majority of human tissues and cell types, which suggests that 1,25(OH)_2_D_3_ has a far more pleiotropic functional profile than the control of calcium resorption in the intestine. In fact, the transcriptome of a variety of cell types showed statistically significant up- and downregulation of a few hundred target genes after stimulation with 1,25(OH)_2_D_3_ [10,11]. However, these investigations (i) were performed mostly in cancer cell lines and not in primary tissues, (ii) represented in vitro treatment with pharmacological doses of 1,25(OH)_2_D_3_ (10 nM or more) and not in vivo supplementation with regular amounts of vitamin D_3_ and/or (iii) were performed in species other than humans [12].

The vitamin D_3_ intervention studies VitDbol (NCT02063334, ClinicalTrials.gov (accessed on 30 August 2023)) [13] and VitDHiD (NCT03537027) [14] investigated which genes respond to vitamin D_3_ supplementation in an in vivo setting in healthy humans. VitDHiD investigated the transcriptome of PBMCs (peripheral blood mononuclear cells), which were obtained from 25 healthy individuals directly before and 24 h after taking an oral vitamin D_3_ bolus (80,000 IU). This represents a monthly supplementation dose and was already applied in the VitDbol trial. Moreover, randomized controlled trials like ViDA (Vitamin D Assessment), which aimed to demonstrate the prevention of cardiovascular events and mortality, administered a bolus of 100,000 IU vitamin D_3_ to more than 5000 adults over a period of 3.3 years [15]. The design of this experiment resembled in vitro stimulations with 1,25(OH)_2_D_3_ [16], but the treatment was entirely in vivo using physiological concentrations of vitamin D_3_ that were converted endogenously to 1,25(OH)_2_D_3_. RNA-sequencing (RNA-seq) analysis indicated 442 protein-coding genes that were significantly (FDR, false discovery rate < 0.05) regulated in PBMCs 24 h after vitamin D_3_ supplementation [14]. In this study, we inspected 88 of these vitamin D target genes for their contribution to 16 different signal transduction pathways. Half of the investigated signal transduction cascades were found to include approximately equal numbers of up- and downregulated vitamin D target genes, but the pathways of HIF1, TNF, MAPK and NFκB were primarily downregulated.

## 2. Results

### 2.1. Functional Annotation of Vitamin D Targets of the VitDHiD Study

**Figure 1:**
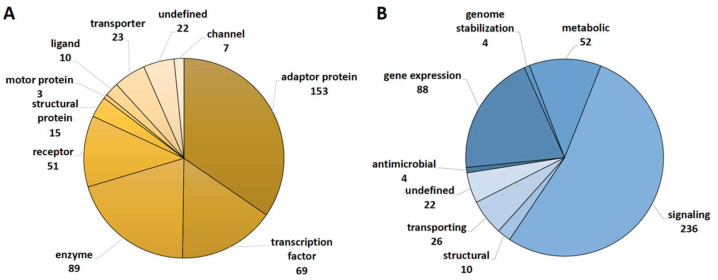
Main roles of vitamin D targets. The databases Human Protein Atlas and GeneCards were used to classify 442 vitamin D target genes identified in the VitDHiD study (Table S1) according to the type of protein that they encode (**A**) and their main biological function (**B**).

In the vitamin D_3_ intervention trial VitDHiD (25 healthy individuals) [14], 442 protein-coding genes were identified as significant (FDR < 0.05) targets of vitamin D in PBMCs that were collected from individuals directly before (day 0 (d0)) and 24 h after (day 1 (d1)) administration of a bolus supplementation of 80,000 IU vitamin D_3_ (Table S1). With the help of the databases Human Protein Atlas (www.proteinatlas.org (accessed on 25 July 2023)) and GeneCards (www.genecards.org (accessed on 23 July 2023)), we functionally annotated the encoded proteins of all target genes with the type of encoded protein (Figure 1A) and primary biological function (Figure 1B). Interestingly, 283 of the 442 target genes encode ligands, receptors, adaptor proteins or transcription factors. In addition, several of the 89 enzymes are cytosolic kinases. This clearly indicates that vitamin D modulates the expression of components of signal transduction cascades. This conclusion is backed up by the observations that 236 target gene products are related to signaling and 88 are involved in gene expression.

### 2.2. Vitamin D Target Genes Involved in KEGG Signal Transduction Pathways

**Figure 2:**
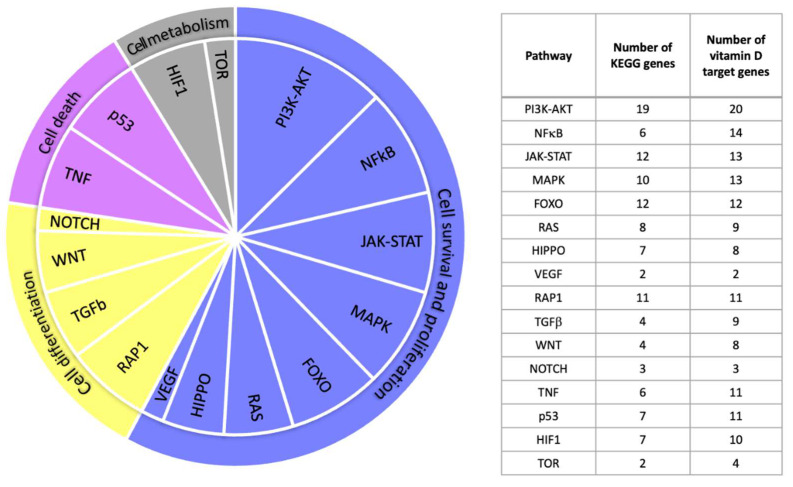
Vitamin D target genes in signal transduction pathways. Proteins encoded by vitamin D target genes were found in 16 signal transduction pathways listed in KEGG (**left**). The number of vitamin D-responsive genes/proteins listed directly in the KEGG pathways is increased by related proteins found in the pool of 442 targets (**right**). Please note that the pathways have some overlapping proteins.

Next, we inspected the signal transduction pathways listed in the KEGG database (www.genome.jp/kegg (accessed on 24 July 2023)) and determined whether any of the 442 vitamin D targets are part of them or represent a closely related protein. In 16 of these signal transduction cascades, we found a total of 88 proteins encoded by vitamin D target genes, of which 64 are directly named in KEGG and 24 are associated (Table S1). The 16 pathways can be categorized into “cell survival and proliferation”, “cellular differentiation”, “cell death” and “cell metabolism” and contain between 2 and 20 vitamin D targets (Figure 2).

**Figure 3:**
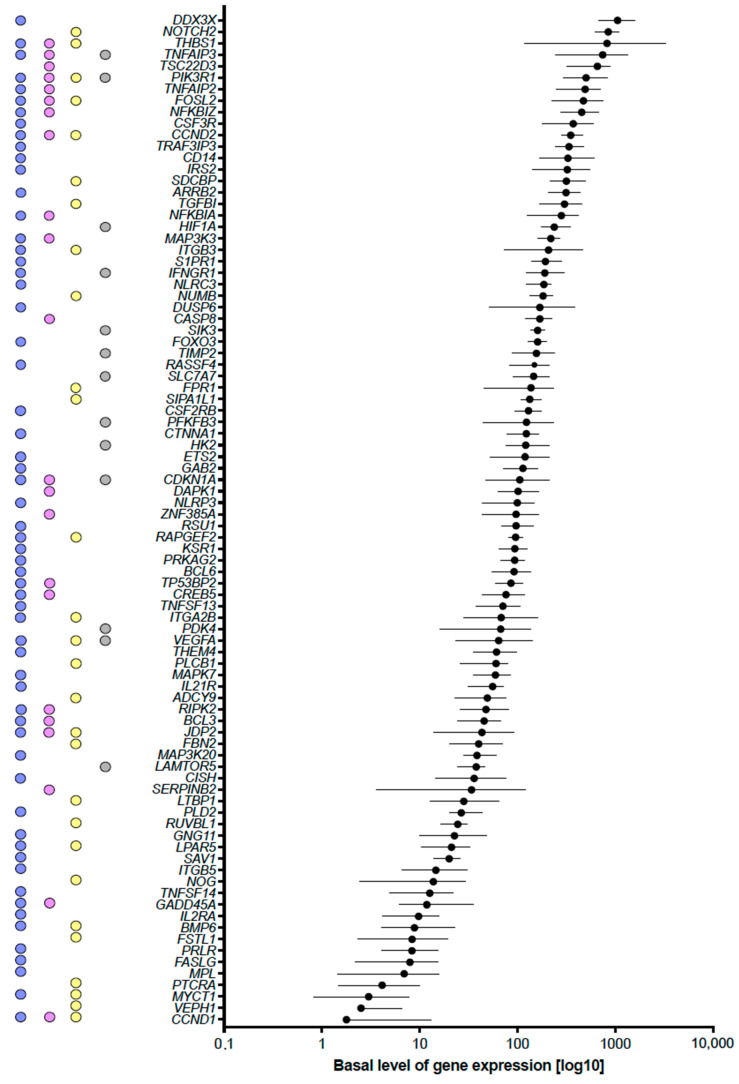
Range of basal expression of vitamin D target genes involved in signal transduction pathways. Colored dots indicate the association of the genes with the four different categories of signal transduction pathways (see Figure 2). Mean basal gene expression (log scale) for the 25 individuals is indicated by black dots, and the range is indicated by horizonal lines.

The 88 vitamin D target genes involved in the KEGG signal transduction pathways show a wide range of basal expression; for example, *DDX3X* (DEAD-box helicase 3 X-linked) exhibits 590-fold higher expression than *CCND1* (cyclin D1) (Figure 3). Furthermore, in the 25 VitDHiD participants, the basal expression of the individual genes ranges from only 1.38-fold for *RAPGEF2* (Rap guanine nucleotide exchange factor 2) to 33.61-fold for *SERPINB2* (serpin family B member 2) (Table S1). In addition, the color coding shown in Figure 3 illustrates that many of the vitamin D target genes are involved in more than one pathway category.

### 2.3. Vitamin D-Triggered Homeostasis

**Figure 4:**
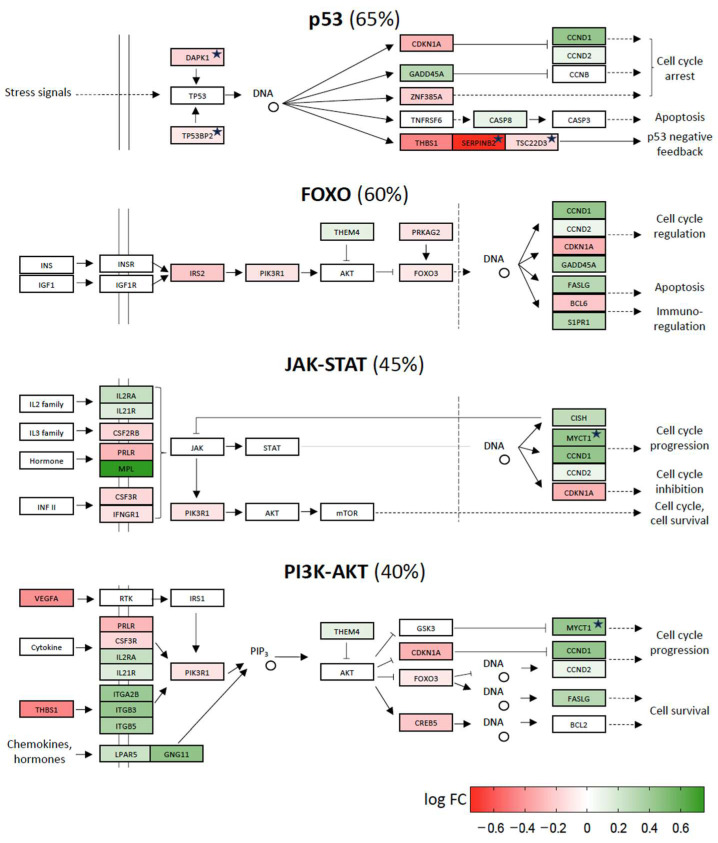
Vitamin D-triggered homeostasis. The signal transduction pathways p53, FOXO, JAK-STAT and PI3K-STAT have approximately equal numbers of protein components, the genes of which are upregulated (green) and downregulated (red) by vitamin D supplementation in healthy individuals. In each pathway the given percentage reflects the number of genes, the direction of regulation of which will contribute to signal inhibition. The color intensity is proportional to the log fold change (FC) of gene expression between d1 and d0. The structure of the pathway follows the design of KEGG. Functionally similar proteins not indicated in KEGG are marked by an asterisk.

When scoring the 16 different pathways for relative numbers of up- and downregulated vitamin D target genes, half of them showed an approximately even count, i.e., between 35 and 65% downregulated genes. Some of the best-known signaling pathways, which are named after their key proteins, such as p53 (tumor protein 53), FOXO (Forkhead box O), JAK-STAT (Janus kinase/signal transducers and activators of transcription) and PI3K-AKT (phosphatidylinositol-3-kinase-AKT serine/threonine kinase), belong to the latter (Figure 4). Interestingly, the same four pathways were indicated as the top scoring pathways by EnrichR analysis (https://maayanlab.cloud/Enrichr (accessed on 28 August 2023)). Furthermore, the pathways of RAP1 (Ras-related protein 1), WNT (Wingless-related integration site), HIPPO (named after the “big headed” phenotype of mutant flies) and NOTCH (named after mutant flies having a “notched” pattern) also have approximately the same number of up- and downregulated vitamin D target genes (Figure S1). Accordingly, the pathways seem to be stabilized by vitamin D so that the cells stay in homeostasis.

### 2.4. Repression of Stress Response Pathways by Vitamin D

**Figure 5:**
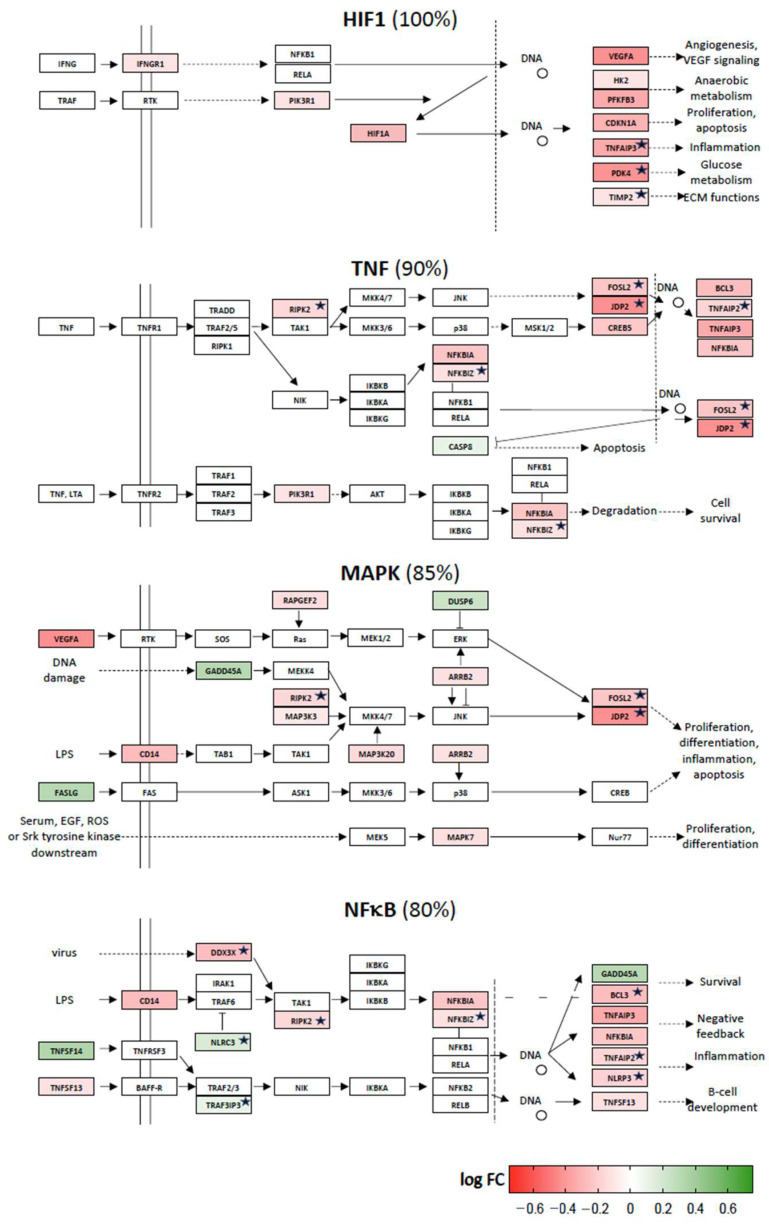
Vitamin D-triggered repression of stress signaling. The signal transduction pathways HIF1, TNF, MAPK and NFκB have more than 75% of their vitamin D target genes downregulated (red), i.e., only less than 25% of the genes are upregulated (green). In each pathway the given percentage reflects the number of genes, the direction of regulation of which will contribute to signal inhibition. The color intensity is proportional to the log fold change (FC) of gene expression between d1 and d0. The structure of the pathway follows the design of KEGG. Functionally similar proteins not indicated in KEGG are marked by an asterisk.

The other eight signaling pathways have 75% or more of their vitamin D target genes downregulated. These are the signal transduction cascades named for the proteins HIF1, TNF, MAPKs and NFκB (Figure 5), as well as those named for VEGF (vascular endothelial growth factor), TGFβ (tumor growth factor β), RAS (rat sarcoma virus) and TOR (target of rapamycin) (Figure S2).

## 3. Discussion

This study was performed in order to evaluate the functional consequences of the up- and downregulation of 442 protein-coding genes that were previously identified in an in vivo study, in which 25 individuals were exposed for 24 h to a vitamin D_3_ bolus (VitDHiD study [14]). A fast and convenient way to evaluate the overall function of a set of target genes is through the use of web services, such as EnrichR [17]. The algorithms behind these services provide a ranking of the most enriched signal transduction pathways based on publicly accessible databases, and KEGG may be the most important and reliable [18]. A KEGG-based EnrichR search using the 442 vitamin D target genes of VitDHiD indicated the signal transduction cascades of FOXO, JAK-STAT, PI3K-AKT and p53, representing best the list of vitamin D target genes, i.e., out of the 16 pathways inspected, they were the only pathways passing the threshold of FDR < 0.05 in the enrichment analysis. Our manual inspection of the pathways confirms that 7 to 19 of the vitamin D target genes are listed in KEGG as components of these very well-known signal transduction pathways. When we enriched the original description of the pathways for genes/proteins that we found as family members and/or interaction partners of the proteins listed in KEGG, 11 to 20 target genes were identified as components of the four pathways. These signal transduction cascades play a key role in the cellular response to the peptide hormone insulin (FOXO and PI3K-AKT), a number of different cytokines (JAK-STAT) and various measures of cellular stress, such as DNA damage and cell cycle arrest (p53). Thus, the first conclusion is that in the immune cells of healthy individuals, vitamin D modulates these important pathways and their associated functions. However, automated analysis via EnrichR does not indicate whether the pathways are up- or downregulated.

Our manual inspection of the FOXO, JAK-STAT, PI3K-AKT and p53 pathways indicated that they contain an approximately even percentage of genes, the direction of regulation of which leads to inhibition and activation of signaling through a given pathway. Thus, the pathways are neither enhanced nor repressed but rather stabilized by vitamin D_3_ supplementation. This makes sense since in healthy individuals, an external, overall beneficial signal, such as vitamin D stimulation, should contribute to the homeostasis of the immune cells rather than inducing any stress [19]. We draw the same conclusion from checking the pathways of RAP1, WNT, HIPPO and NOTCH, i.e., vitamin D treatment stabilizes them. In contrast, the examination of eight additional KEGG pathways indicated that 75 to 100% of the vitamin D targets found in them are downregulated. Thus, the activity of these pathways is reduced by vitamin D_3_ supplementation. Interestingly, of the 16 evaluated KEGG signal transduction pathways, none of them are enhanced by vitamin D.

The common characteristic of the eight downregulated KEGG signal transduction pathways is that they represent responses to different types of stress, such as oxygen deprivation (HIF1 and VEGF), microbe infection (TNF), growth stimulation (MAPK, TGFβ and RAS), inflammation (NFκB) and protein overload (TOR). Accordingly, supplementation with vitamin D_3_ reduces stress on immune cells, which should increase their survival and prevent possible overreactions. This conclusion fits with the observation that vitamin D is an important modulator of adaptive immunity, leading to the prevention of autoimmune diseases [20]. In contrast, vitamin D insufficiency is linked to a large set of immunologic disorders, such as multiple sclerosis [21], inflammatory bowel disease [22], rheumatoid arthritis [23], type I diabetes [24] and infections like tuberculosis [25].

The 16 signal transduction pathways investigated in this study are not independent from each other but have a number of key proteins in common. Some of these common proteins, such as VEGF, CCND1, GADD45A (growth arrest and DNA damage-inducible alpha) and THBS1 (thrombospondin 1), are encoded by vitamin D target genes. Equal numbers of these genes are up- and downregulated, which supports our statement that vitamin D mediates the homeostasis of cells rather than inducing polarization.

Some of the investigated genes have already been described as vitamin D targets. For example, *GADD45A* is a vitamin D target gene in the context of prostate cancer cells [26], and its involvement in MAPK signaling has been reported in colon cancer cells [27]. However, we demonstrate that the *GADD45A* gene is regulated in an human in vivo setting of healthy immune cells. Moreover, the design of the VitDHiD study, which included a measurement as early as 24 h after vitamin D_3_ supplementation, allows a better comparison to the above-mentioned in vitro studies in cancer cell lines.

The vitamin D target genes involved in signal transduction cascades have a wide range of basal expression. Most of the highly expressed genes, such as *THBS1*, are downregulated by vitamin D_3_ supplementation, while lowly expressed genes, such as *CCND1*, tend to be upregulated. Both genes had previously been described as vitamin D targets [28]. Interestingly, these gene examples also show a large interindividual variation in their basal expression and inducibility. This suggests that the impact of vitamin D_3_ supplementation in healthy individuals can differ from person to person. This fits with our observation of a vitamin D response index, which segregates individuals as high, mid and low responders to vitamin D [29]. Therefore, interindividual differences in the vitamin D response index may be explained by variations in these genes and the pathways mediated by them.

This study has a few limitations. The number of genes reliably related to signal transduction pathways is still limited, i.e., KEGG assigns only 20% of all human genes to pathways to date [18]. Accordingly, our conclusions are based only on 88 of the 442 identified vitamin D target genes. Moreover, present knowledge on the in vivo actions of vitamin D is primarily based on studying diseases and/or vitamin D deficiency. At present, there are no studies comparable to the design of the VitDHiD study, so our results cannot be directly backed up. For example, the study of Hossein-nezhad et al. [30] also investigated PBMCs from healthy individuals, but it was designed as an 8-week trial with daily vitamin D_3_ supplementation. However, it is a common observation that sufficient vitamin D_3_ supplementation improves the health status of individuals [31].

In conclusion, based on manual inspection of the role of 88 in vivo vitamin D target genes in 16 KEGG signal transduction pathways, vitamin D either stabilizes or inhibits them. In this way, vitamin D contributes to the homeostasis of the immune system of healthy individuals or reduces cellular stress, respectively.

## 4. Materials and Methods

### 4.1. VitDHiD Trial

The VitDHiD trial (NCT03537027) was performed in May 2018 using healthy students and staff (12 females and 13 males, age 21–54, body mass index 21.4–25.6 and total basal 25(OH)D_3_ levels 39.0–124.5 nM) of the University of Eastern Finland in Kuopio. The major aim of the study was to investigate the vitamin D response index of the individuals by comparing PBMCs at d0 before an oral vitamin D_3_ bolus (80,000 IU) and 24 h later (d1) [14]. All research was performed in accordance with relevant guidelines and regulations. All participants gave written informed consent to participate in the study.

### 4.2. Identification of Vitamin D Target Genes

The detailed analysis of vitamin D target genes will be described elsewhere. In brief, PBMCs were isolated within one hour after a blood draw from 20 mL of peripheral blood in Vacutainer CPT Cell Preparation Tubes with sodium citrate (Becton Dickinson, Eysins, Switzerland) according to the manufacturer’s instructions. Total RNA was extracted using the High Pure RNA Isolation kit (Roche Diagnostics, Penzberg, Germany) following the manufacturer’s protocol. RNA quality was assessed on an Agilent Bioanalyzer, and library preparation was performed after rRNA depletion applying kits and protocols from New England Biolabs (Ipswich, MA, USA). RNA-seq libraries were sequenced at a 75 bp read length on a NextSeq 500 system (Illumina, San Diego, CA, USA) using standard protocols at the Gene Core of the EMBL (Heidelberg, Germany). Differential gene expression was computed using *DESeq2* [32] and *EdgeR* [33], both of which implement a negative binomial test over the reads in the two conditions (d1/d0), with standard parameters and a FDR cutoff of 0.05. Only protein-coding genes were considered. This resulted in 442 genes, which are listed in Table S1.

### 4.3. Classification of Vitamin D Target Genes

All 442 vitamin D target genes (Table S1) were inspected manually for their function, such as type of encoded protein and its primary biological function, with the help of databases such as Human Protein Atlas and GeneCards. Information on the involvement of a subset of the vitamin D target genes in signal transduction pathways relies on KEGG and information from the literature.

## Data Availability

All data relevant to this study can be found in Table S1.

## Supplementary Materials

The following files are available online at https://www.mdpi.com/article/10.3390/ijms241914632/s1.
