# Supplementary material for: In Vivo Regulation of Signal Transduction Pathways by Vitamin D Stabilizes Homeostasis of Human Immune Cells and Counteracts Molecular Stress
Source: Int J Mol Sci. 2023 Sep 27;24(19):14632. doi: 10.3390/ijms241914632 (PMC10572664; doi:10.3390/ijms241914632)
Supplement: Supplementary file 1 [file ijms-24-14632-s001.zip › ijms-2611921-supplementary/Figures S1 and S2.pdf]

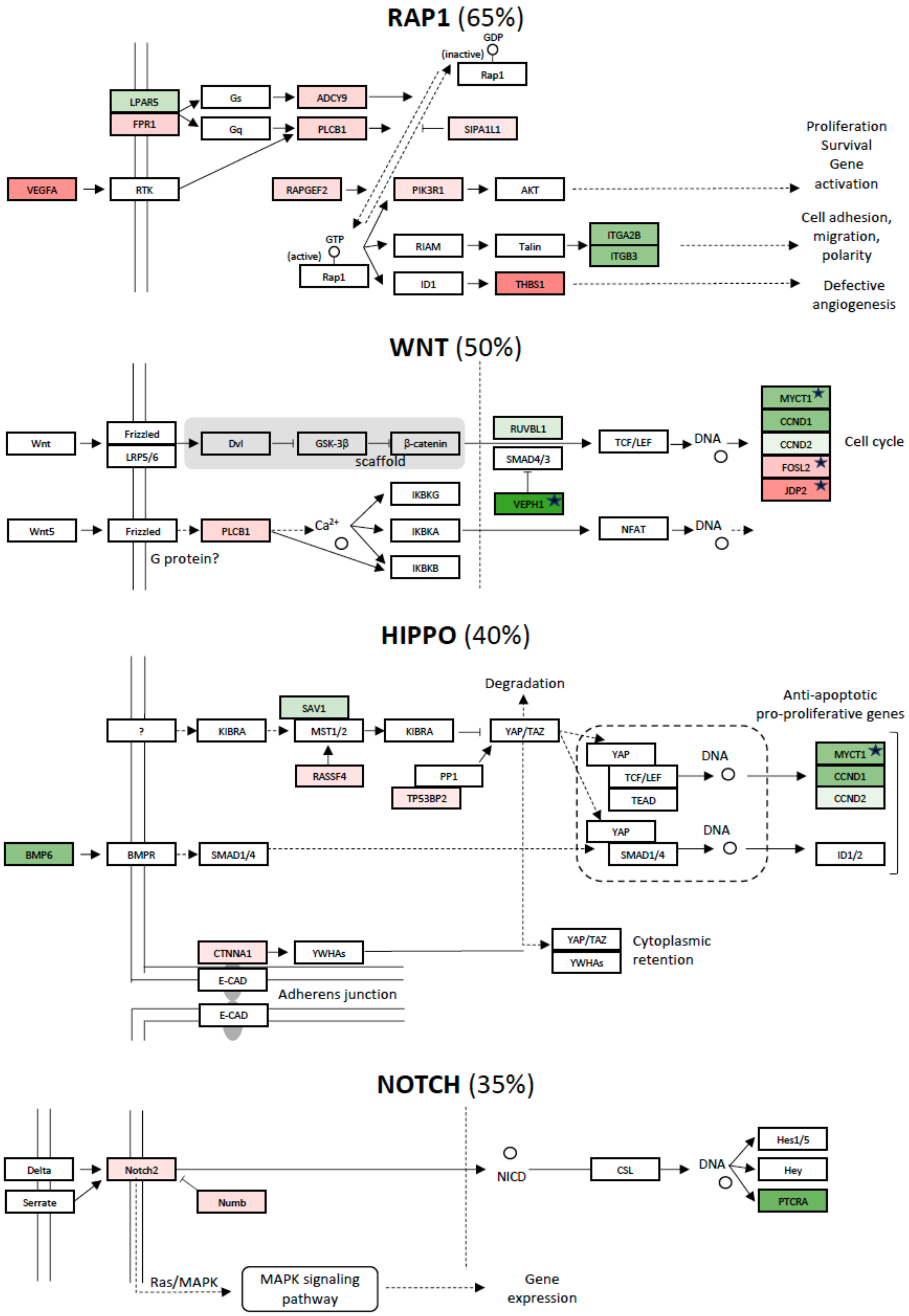

Figure S1. The pathways RAP1, WNT, HIPPO and NOTCH. The four indicated signal transduction pathways have an about equal number of protein components, the genes of which are upregulated (green) and downregulated (red) by vitamin D supplementation of healthy individuals. The given percentage reflects the number of genes, whose direction of regulation will contribute to signal inhibition in each pathway. Color intensity is proportional to log fold change (FC) of gene expression between d1 and d0. The structure of the pathway follows the design of KEGG. Functionally similar proteins not indicated in KEGG are marked by an asterisk.

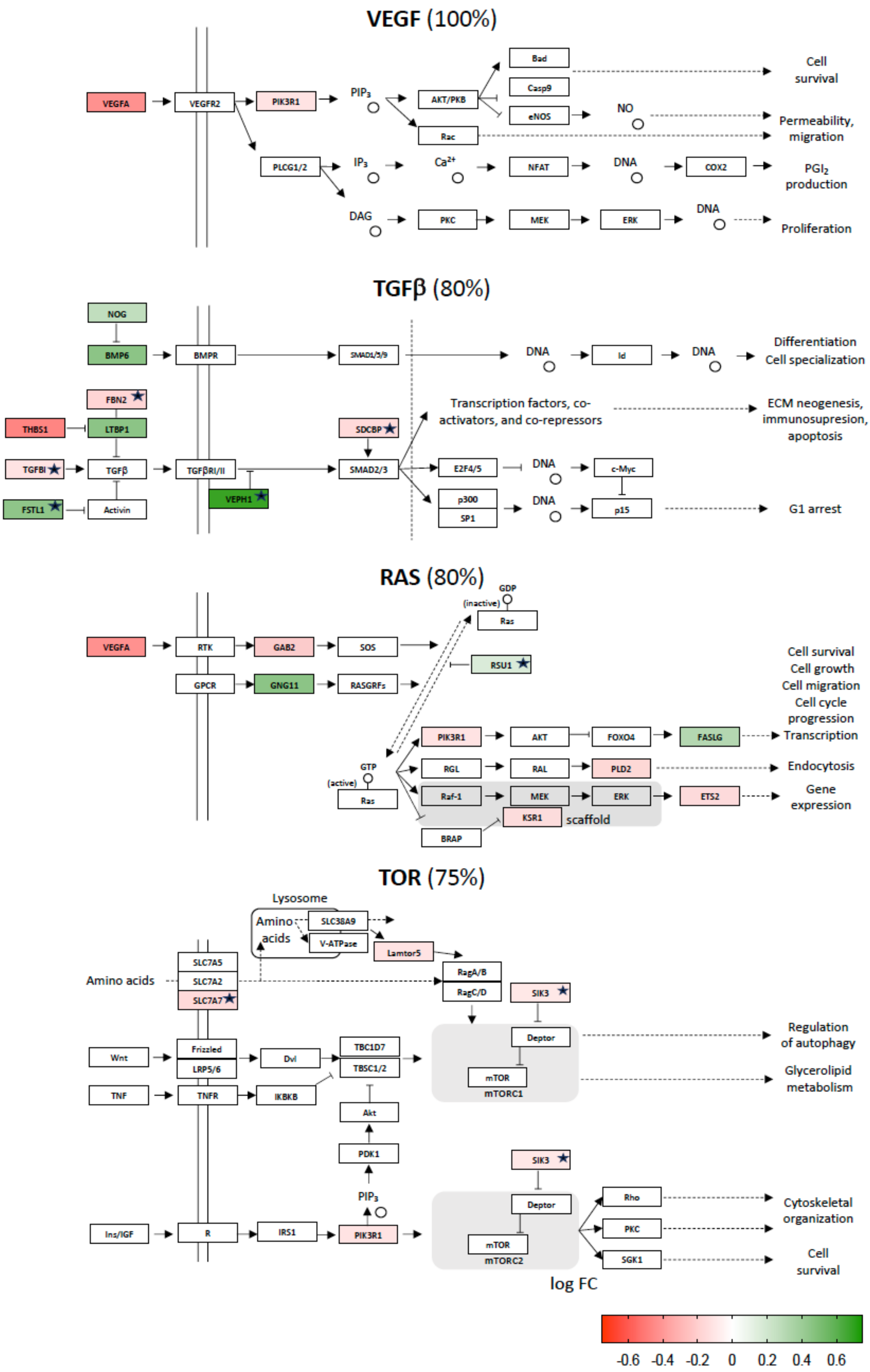

Figure S2. The pathways VEGF, TGFb, RAS and TOR. The four indicated signal transduction pathways have more than 75% of their vitamin D target genes downregulated (red), *i.e.*, only less than 25% of the genes are upregulated (green). The given percentage reflects the number of genes, whose direction of regulation will contribute to signal inhibition in each pathway. Color intensity is proportional to log fold change (FC) of gene expression between d1 and d0. The structure of the pathway follows the design of KEGG. Functionally similar proteins not indicated in KEGG are marked by an asterisk.
